# Sex-Specific Cardiovascular Phenotypes in Marfan Syndrome

**DOI:** 10.1016/j.jacadv.2026.103034

**Published:** 2026-07-20

**Authors:** Luke Dreher, Hussein Abdul Nabi, Hunter B. VanDolah, Jack Hartnett, Yuxiang Wang, Christine Firth, Linnea M. Baudhuin, Hicham El Masry, Chadi Ayoub, Fadi Shamoun

**Affiliations:** a Department of Cardiovascular Medicine, Mayo Clinic Arizona, USA; b Department of Laboratory Medicine and Pathology, Mayo Clinic, Rochester, Minnesota, USA; c Department of Clinical Genomics, Mayo Clinic, Rochester, Minnesota, USA

**Keywords:** aneurysm, aortic aneurysm, arrhythmias, connective tissue disorder, Marfan syndrome, sex differences

## Abstract

**Background:**

Marfan syndrome (MFS) is a heritable connective tissue disorder in which cardiovascular complications drive morbidity. Contemporary sex-based differences in vascular and rhythm manifestations remain incompletely defined.

**Objectives:**

The purpose of this study was to compare aneurysm burden, dissection prevalence, arrhythmias, valvular phenotypes, and cardiovascular interventions between men and women with confirmed MFS.

**Methods:**

We performed a retrospective cohort study of adults with confirmed MFS evaluated from 2018 to 2024 at Mayo Clinic. Diagnoses and vascular outcomes were confirmed by manual chart and imaging report review. Outcomes reflected ever-documented history during the study period. Multivariable logistic regression estimated sex-based associations adjusted for age, hypertension, diabetes mellitus, chronic kidney disease, and heart failure. Sensitivity analyses were performed in the genetically positive subgroup.

**Results:**

Among 783 patients (443 men, 340 women), men more often had ascending aortic aneurysm (89.1% vs 71.1%), any aneurysm (91.0% vs 75.3%), and any extra-aortic aneurysm (36.8% vs 25.9%) (all *P* ≤ 0.001). Dissection prevalence was similar by sex. Men also had more atrial fibrillation (44.0% vs 30.9%), atrial flutter (18.3% vs 12.1%), sustained ventricular tachycardia (9.7% vs 5.0%), and aortic root surgery (63.4% vs 40.3%) (all *P* < 0.05), whereas women more often had mitral valve prolapse (45.6% vs 37.7%; *P* = 0.028). After adjustment, male sex remained associated with aneurysmal burden, atrial fibrillation, sustained ventricular tachycardia, and aortic root surgery.

**Conclusions:**

In this multicenter Marfan cohort, men had a greater burden of aneurysmal disease, atrial arrhythmias, sustained ventricular tachycardia, and aortic intervention, whereas dissection prevalence was similar between sexes.

Marfan syndrome (MFS) is a connective tissue disorder characterized by multisystem involvement, with cardinal features affecting the skeletal, ocular, and cardiovascular systems.^1^, ^2^, ^3^ Within the broader category of heritable thoracic aortic disease, nearly a quarter of patients fall within a defined syndrome such as Marfan or Loeys-Dietz.^4^^,^^5^ Patients with MFS face a disproportionately high burden of adverse health outcomes, with a shorter life expectancy stemming largely from aortic and other cardiovascular causes. However, with proper medical management, life expectancy is now approaching that of the general population.^6^, ^7^, ^8^, ^9^, ^10^

While several retrospective studies have demonstrated no statistically significant difference in mortality between males and females with MFS, males consistently experience a higher burden of worse cardiovascular outcomes compared to their female counterparts. This disparity appears to be driven primarily by increased rates of aortic events and a greater need for prophylactic surgical intervention, based on increased risk of death or aortic dissection at absolute aortic diameters ≥50 mm.^11^, ^12^, ^13^, ^14^, ^15^, ^16^ Additionally, the aortic events tend to occur at younger ages in male patients.^9^^,^^13^ It has been suggested that estrogen may have a protective effect, supported by observations of worsening aortic aneurysm progression during pregnancy.^6^^,^^17^ Additionally, murine models suggest estrogen can lead to enhanced fibrillin-1 production and less aortic wall damage, along with greater aortic stiffness observed in male mice compared to female mice in severe MFS models.^18^, ^19^, ^20^ Specific *FBN1* variants resulting in haploinsufficiency were shown to have a higher risk of dissection or death regardless of sex.^9^^,^^21^^,^^22^ Despite these differences, the overall risk of major cardiovascular events remains high for both sexes as they age.^14^ This ongoing risk is reflected by the continued development of extrathoracic aneurysmal disease as well as arrhythmias, even among patients without prior cardiovascular surgery or valvular disease.^19^^,^^23^, ^24^, ^25^, ^26^

While larger national registries have provided additional evidence regarding aortic and extra-aortic cardiovascular manifestations of MFS, data on sex-based differences in cardiovascular manifestations and subsequent interventions remain incompletely characterized. This study aimed to further evaluate sex-specific differences in comorbidities, aneurysm and dissection distribution, arrhythmias, valvular abnormalities, and cardiovascular interventions in patients with MFS.

## Methods

### Study population

This retrospective cohort study included adult patients (≥18 years) evaluated for MFS between 2018 and 2024 across Mayo Clinic sites in Rochester, Arizona, and Florida. Patients were initially identified using International Classification of Diseases-Tenth Revision codes, and all potential cases underwent manual chart review for diagnostic confirmation. Of 1,113 patients screened, 783 met inclusion criteria after adjudication. Among these, 353 had a genetically confirmed diagnosis with documented pathogenic or likely pathogenic *FBN1* variants; the remainder were diagnosed phenotypically by retrospective chart adjudication using revised Ghent criteria, including cases supported by family history and compatible clinical features.^1^ Patients with unclear diagnostic features or unresolved suspicion for alternative connective tissue disorders, including other heritable thoracic aortic diseases such as Loeys-Dietz syndrome, were excluded. The study was approved as exempt by the Institutional Review Board, and informed consent was waived due to its retrospective design.

### Data collection

Comprehensive manual chart review was performed to abstract demographics, comorbidities, arrhythmia and conduction history, valvular disease, aortic and extra-aortic aneurysms/dissections, cardiovascular interventions, and selected event-timing variables. Overall analyses focused on sex-based differences in documented prevalence across the medical record. Selected timing variables were additionally abstracted where reliably available for prespecified outcomes, including age at atrial fibrillation (AF) diagnosis, age at first ventricular fibrillation (VF), and age at first cardiac surgery. Cardiac procedures were recorded as present/absent and included aortic root replacement; aortic, mitral, and tricuspid valve repair or replacement; and patent foramen ovale closure.

Arrhythmias included AF, atrial flutter (AFL), supraventricular tachycardia (SVT), ventricular tachycardia (VT) categorized as sustained or nonsustained, VF, and conduction abnormalities. SVT was defined as clinician-documented non-AF, non-AFL supraventricular tachyarrhythmias, including atrial tachycardia, atrioventricular nodal reentrant tachycardia, atrioventricular reentrant tachycardia, and other specified SVTs. Nonsustained VT was defined as ≥3 consecutive beats lasting <30 seconds with spontaneous termination.^27^

Echocardiographic data were abstracted from available studies. Left ventricular ejection fraction (LVEF) was recorded as the lowest documented value, using 3-dimensional assessment when available or 2-dimensional biplane measurement otherwise.^28^ Valvular abnormalities, including mitral, tricuspid, and aortic regurgitation, mitral valve prolapse, aortic stenosis, and bicuspid aortic valve, were recorded using the most recent documented severity assessment; when valve or aortic surgery had occurred, the preoperative echocardiogram was preferentially used.

Aneurysms and dissections were adjudicated by manual review of imaging reports, including computed tomography, magnetic resonance imaging, transthoracic echocardiography for ascending aortic aneurysm, and abdominal ultrasound for abdominal aortic aneurysm. Ascending aortic aneurysm was defined as an ascending aortic measurement exceeding the age-, sex-, and body surface area-adjusted upper-limit threshold in the Mayo Clinic Enterprise Imaging Management System when such measurements were available. When threshold-based measurement data were unavailable, ascending aortic aneurysm was classified based on clear radiology-report interpretation or supporting clinical documentation. To provide clinical context for this threshold-based classification, absolute and body surface area (BSA)-indexed aortic root/sinus and tubular ascending diameters, as well as BSA-based upper-limit root thresholds, were summarized among patients with and without ascending aortic aneurysm. Abdominal and extra-aortic aneurysms/dissections were classified based on radiology report interpretation and supporting clinical documentation, as uniform retrospective diameter extraction across abdominal and extra-aortic vascular territories was not consistently feasible. Vascular imaging was obtained for clinical indications rather than by standardized research protocol; therefore, imaging completeness varied across vascular beds, and prevalence estimates reflect documented findings among imaged territories.

### Study outcomes

The primary objective was to evaluate sex-based differences in clinical characteristics, cardiovascular manifestations, and interventions among patients with MFS. Outcomes compared between men and women included comorbidities, conduction abnormalities, aortic and extra-aortic aneurysms, dissections, valvular abnormalities, and cardiovascular procedures. All outcomes were defined as a history of the condition ever documented in the electronic health record during the study period and were analyzed as binary variables (present vs absent). In addition to these prevalence-based outcomes, selected age-at-event variables were examined for prespecified outcomes when reliably available, including age at AF diagnosis, age at first VF, and age at first cardiac surgery.

### Statistical analysis

All analyses were performed using Python (version 3.13). Categorical variables are presented as counts (percentages) and continuous variables as mean ± SD or median (IQR) according to distribution assessed by the Shapiro-Wilk test. Sex-based comparisons used chi-square tests (or Fisher exact test when expected cell counts were small) for categorical variables, Student’s *t*-test for normally distributed continuous variables, and Mann-Whitney *U* test for nonnormally distributed continuous variables. Two-tailed *P* values <0.05 were considered statistically significant. Univariate logistic regression models estimated associations between sex and each binary outcome, reported as ORs with 95% CIs. Multivariable logistic regression models were constructed for prespecified vascular, arrhythmic/conduction, valvular, and procedural outcomes (listed in the Supplement) to estimate sex-based associations adjusted for age, hypertension, diabetes mellitus (DM), chronic kidney disease (CKD), and heart failure (HF). Descriptive analyses used available data for each variable, with row-specific denominators reported where applicable. Multivariable models used complete-case analysis without imputation, and model-specific sample sizes reported alongside the corresponding results. Supplemental Tables 4A to 4F. To limit collinearity, closely related variables were not included simultaneously within the same model (eg, continuous LVEF vs LVEF <40%). Prespecified sensitivity analyses were repeated in the genetically positive subgroup, defined by documented pathogenic or likely pathogenic *FBN1* variants (n = 353).

**Table 1:** Baseline Characteristics of Marfan Syndrome Stratified by Sex

	Overall Cohort (N = 783)	Male (n = 443)	Female (n = 340)	*P* Value
Age, y (mean ± SD)	52.6 ± 15.2	52.1 ± 15.2	53.2 ± 15.2	0.333
Follow-up time, y (mean ± SD)	12.7 ± 7.6	12.6 ± 7.7	12.8 ± 7.4	0.748
Genetically positive, n (%)	353 (45.1%)	186 (42.0%)	167 (49.1%)	0.051
Left ventricular EF (%), mean ± SD	56.6 ± 10.8	56.3 ± 11.1	57.0 ± 10.4	0.315
Hypertension, n (%)	348 (44.4%)	218 (49.2%)	130 (38.2%)	0.002
Chronic kidney disease, n (%)	91 (11.6%)	61 (13.8%)	30 (8.8%)	0.033
Migraines, n (%)	116 (14.8%)	45 (10.2%)	71 (20.9%)	<0.001
Stroke or TIA, n (%)	58 (7.4%)	40 (9.0%)	18 (5.3%)	0.054
Heart failure, n (%)	136 (17.4%)	84 (19.0%)	52 (15.3%)	0.184
Diabetes mellitus, n (%)	46 (5.9%)	32 (7.2%)	14 (4.1%)	0.091

Values are n (%) unless otherwise specified. Age and left ventricular ejection fraction (EF) are presented as mean ± SD. *P* values reflect sex-based comparisons using chi-square tests (or Fisher exact test when expected cell counts were small) for categorical variables and parametric or nonparametric tests as appropriate for continuous variables. Two-tailed *P* values <0.05 were considered statistically significant. EF was recorded using 3-dimensional assessment when available or 2-dimensional biplane measurement otherwise.

EF = ejection fraction; TIA = transient ischemic attack.

**Table 2:** Aneurysm and Dissection Prevalence in Marfan Syndrome Stratified by Sex

	Overall Cohort (N = 783)	Male (n = 443)	Female (n = 340)	*P* Value
Aneurysms				
Ascending aortic, n/N (%)	635/781 (81.3%)	394/442 (89.1%)	241/339 (71.1%)	<0.001
Abdominal aortic, n (%)	179 (22.9%)	120 (27.1%)	59 (17.4%)	0.001
Any aneurysm, n (%)	659 (84.2%)	403 (91.0%)	256 (75.3%)	<0.001
Any extra-aortic aneurysm, n (%)	251 (32.1%)	163 (36.8%)	88 (25.9%)	0.001
Subclavian, n (%)	39 (5.0%)	24 (5.4%)	15 (4.4%)	0.620
Cerebral, n (%)	27 (3.4%)	17 (3.8%)	10 (2.9%)	0.557
Carotid, n (%)	57 (7.3%)	32 (7.2%)	25 (7.4%)	1.000
Vertebral, n (%)	26 (3.3%)	13 (2.9%)	13 (3.8%)	0.549
Pulmonary, n (%)	34 (4.3%)	16 (3.6%)	18 (5.3%)	0.290
Coronary, n (%)	29 (3.7%)	21 (4.7%)	8 (2.4%)	0.088
Splenic, n (%)	8 (1.0%)	5 (1.1%)	3 (0.9%)	1.000
Mesenteric, n (%)	23 (2.9%)	17 (3.8%)	6 (1.8%)	0.133
Celiac, n (%)	4 (0.5%)	3 (0.7%)	1 (0.3%)	0.637
Renal, n (%)	18 (2.3%)	12 (2.7%)	6 (1.8%)	0.474
Hepatic, n (%)	5 (0.6%)	3 (0.7%)	2 (0.6%)	1.000
Gastric, n (%)	6 (0.8%)	4 (0.9%)	2 (0.6%)	0.702
Iliac, n (%)	99 (12.6%)	77 (17.4%)	22 (6.5%)	<0.001
Femoral, n (%)	11 (1.4%)	10 (2.3%)	1 (0.3%)	0.028
Popliteal, n (%)	11 (1.4%)	10 (2.3%)	1 (0.3%)	0.028
Axillary, n (%)	7 (0.9%)	5 (1.1%)	2 (0.6%)	0.705
Brachial, n (%)	13 (1.7%)	7 (1.6%)	6 (1.8%)	1.000
Internal mammary, n (%)	7 (0.9%)	5 (1.1%)	2 (0.6%)	0.705
Dissections				
Type A dissection, n (%)	114 (14.6%)	65 (14.7%)	49 (14.4%)	1.000
Type B dissection, n (%)	115 (14.7%)	65 (14.7%)	50 (14.7%)	1.000
Any dissection, n (%)	227 (29.0%)	131 (29.6%)	96 (28.2%)	0.692
Any extra-aortic dissection, n (%)	41 (5.2%)	29 (6.5%)	12 (3.5%)	0.074
Carotid, n (%)	13 (1.7%)	8 (1.8%)	5 (1.5%)	0.785
Vertebral, n (%)	4 (0.5%)	2 (0.5%)	2 (0.6%)	1.000
Subclavian, n (%)	7 (0.9%)	6 (1.4%)	1 (0.3%)	0.146
Coronary, n (%)	6 (0.8%)	3 (0.7%)	3 (0.9%)	1.000
Superior mesenteric, n (%)	3 (0.4%)	3 (0.7%)	0 (0.0%)	0.262
Celiac, n (%)	1 (0.1%)	1 (0.2%)	0 (0.0%)	1.000
Iliac, n (%)	13 (1.7%)	10 (2.3%)	3 (0.9%)	0.165
Femoral, n (%)	2 (0.3%)	1 (0.2%)	1 (0.3%)	1.000
Imaging (MR or CT)				
Head and neck, n (%)	396 (50.6%)	213 (48.1%)	183 (53.8%)	0.113
Chest, n (%)	636 (81.2%)	361 (81.5%)	275 (80.9%)	0.854
Abdomen and pelvis, n (%)	598 (76.4%)	332 (74.9%)	266 (78.2%)	0.309

Ascending aortic aneurysm is presented as n/N (%) using row-specific evaluable denominators because ascending aortic aneurysm status was available in 781 patients overall, including 442 men and 339 women. All other binary outcomes used the full cohort denominator unless otherwise specified. Values are n (%) unless otherwise specified. Aneurysms and dissections were adjudicated by manual review of imaging reports (computed tomography or magnetic resonance imaging; transthoracic echocardiogram for ascending aortic aneurysm; abdominal ultrasound for abdominal aortic aneurysm) with anatomic distribution recorded. *P* values reflect sex-based comparisons using chi-square tests (or Fisher exact test when expected cell counts were small).

**Table 3:** Arrhythmias and Systolic Dysfunction, Overall and by Sex

	Overall Cohort (N = 783)	Male (n = 443)	Female (n = 340)	*P* Value
Atrial fibrillation, n (%)	300 (38.3%)	195 (44.0%)	105 (30.9%)	<0.001
Age at atrial fibrillation diagnosis, y (mean ± SD)	50.4 ± 14.0 (279)	49.1 ± 13.5 (179)	52.9 ± 14.6 (100)	0.034
Atrial flutter, n (%)	122 (15.6%)	81 (18.3%)	41 (12.1%)	0.017
Right bundle branch block, n (%)	117 (14.9%)	79 (17.8%)	38 (11.2%)	0.011
Left bundle branch block, n (%)	54 (6.9%)	36 (8.1%)	18 (5.3%)	0.154
Supraventricular tachycardia, n (%)	136 (17.4%)	69 (15.6%)	67 (19.7%)	0.153
Nonsustained VT, n (%)	95 (12.1%)	59 (13.3%)	36 (10.6%)	0.270
Sustained VT, n (%)	60 (7.7%)	43 (9.7%)	17 (5.0%)	0.015
Ventricular fibrillation, n (%)	41 (5.2%)	30 (6.8%)	11 (3.2%)	0.034
Age at first ventricular fibrillation, y (mean ± SD)	43.1 ± 14.3 (37)	44.1 ± 15.2 (28)	40.0 ± 11.1 (9)	0.383
EF <40%, n (%)	67 (8.6%)	42 (9.5%)	25 (7.4%)	0.306

Values are n (%) unless otherwise specified. Age variables are presented as mean ± SD, with available n shown in parentheses. Arrhythmias reflect a history ever documented in the electronic health record during the study period. Nonsustained ventricular tachycardia (VT) was defined as ≥3 consecutive beats lasting <30 seconds with spontaneous termination. *P* values reflect sex-based comparisons using chi-square tests (or Fisher exact test when expected cell counts were small).

VT = ventricular tachycardia; other abbreviation as in Table 1.

**Table 4:** Valvular Disease Severity and Cardiac Surgery, Overall and by Sex

	Overall Cohort (N = 783)	Male (n = 443)	Female (n = 340)	*P* Value
Valvular disease				
Aortic valve regurgitation				0.282
Mild, n (%)	78 (10.0%)	43 (9.7%)	35 (10.3%)	
Moderate, n (%)	53 (6.8%)	33 (7.4%)	20 (5.9%)	
Severe, n (%)	91 (11.6%)	61 (13.8%)	30 (8.8%)	
Bicuspid aortic valve, n (%)	26 (3.3%)	15 (3.4%)	11 (3.2%)	>0.900
Mitral valve regurgitation				0.291
Mild, n (%)	226 (28.9%)	121 (27.3%)	105 (30.9%)	
Moderate, n (%)	85 (10.9%)	42 (9.5%)	43 (12.6%)	
Severe, n (%)	92 (11.7%)	56 (12.6%)	36 (10.6%)	
Mitral valve prolapse, n (%)	322 (41.1%)	167 (37.7%)	155 (45.6%)	0.028
Tricuspid valve regurgitation				0.593
Mild, n (%)	220 (28.1%)	125 (28.2%)	95 (27.9%)	
Moderate, n (%)	107 (13.7%)	55 (12.4%)	52 (15.3%)	
Severe, n (%)	20 (2.6%)	10 (2.3%)	10 (2.9%)	
Cardiac surgery				
Aortic root replacement or repair, n (%)	418 (53.4%)	281 (63.4%)	137 (40.3%)	<0.001
Aortic valve surgery, n (%)	310 (39.6%)	209 (47.2%)	101 (29.7%)	<0.001
Mitral valve surgery, n (%)	126 (16.1%)	75 (16.9%)	51 (15.0%)	0.493
Tricuspid valve surgery, n (%)	20 (2.6%)	12 (2.7%)	8 (2.4%)	>0.900
Age at first surgery, y (mean ± SD)	36.7 ± 13.8 (455)	36.0 ± 13.7 (299)	38.0 ± 14.0 (156)	0.148

Values are n (%) unless otherwise specified. Age at first surgery is presented as mean ± SD, with available n shown in parentheses. Regurgitation severity categories reflect documented echocardiography grading. *P* values reflect sex-based comparisons using chi-square tests (or Fisher exact test when expected cell counts were small). Cardiac procedures were recorded as present/absent and included aortic root replacement/repair; aortic, mitral, and tricuspid valve repair or replacement (AVR/MVR/TVR).

**Table 5:** Multivariable Associations of Male Sex With Cardiovascular Outcomes in the Overall Cohort and Genetically Positive Subgroup

	n	aOR (95% CI)	*P* Value	GP, n	GP, aOR (95% CI)	GP, *P* Value
Aneurysms and dissections						
Ascending aortic aneurysm	781	3.11 (2.10-4.60)	<0.001	352	4.69 (2.28-9.66)	<0.001
Abdominal aortic aneurysm	783	1.66 (1.14-2.42)	0.008	353	1.80 (1.03-3.14)	0.039
Any aneurysm	783	3.18 (2.09-4.85)	<0.001	353	4.53 (2.13-9.63)	<0.001
Any extra-aortic aneurysm	783	1.65 (1.18-2.29)	0.003	353	1.72 (1.06-2.80)	0.028
Type A dissection	783	0.96 (0.63-1.45)	0.845	353	1.01 (0.55-1.86)	0.983
Type B dissection	783	0.82 (0.54-1.25)	0.356	353	0.62 (0.31-1.21)	0.158
Any dissection	783	0.92 (0.65-1.29)	0.624	353	0.77 (0.46-1.28)	0.310
Any extra-aortic dissection	783	1.74 (0.87-3.52)	0.120	353	1.94 (0.75-5.04)	0.172
Arrhythmias						
Atrial fibrillation	783	1.95 (1.39-2.75)	<0.001	353	1.94 (1.15-3.29)	0.014
Atrial flutter	783	1.57 (1.01-2.44)	0.047	353	1.42 (0.74-2.70)	0.292
Right bundle branch block	783	1.68 (1.10-2.57)	0.017	353	1.73 (0.99-3.03)	0.056
Left bundle branch block	783	1.50 (0.83-2.72)	0.178	353	2.43 (0.98-6.02)	0.055
Supraventricular tachycardia	783	0.73 (0.50-1.07)	0.104	353	0.65 (0.38-1.12)	0.119
Nonsustained VT	783	1.17 (0.73-1.86)	0.509	353	1.37 (0.71-2.63)	0.350
Sustained VT	783	1.84 (1.01-3.35)	0.045	353	2.80 (1.12-7.04)	0.028
Valvular pathology						
Moderate–severe AR	783	1.47 (0.99-2.18)	0.057	353	1.38 (0.77-2.49)	0.278
Moderate–severe TR	783	0.71 (0.47-1.06)	0.093	353	0.59 (0.32-1.09)	0.093
Moderate–severe MR	783	0.90 (0.63-1.30)	0.579	353	0.69 (0.39-1.20)	0.188
Mitral valve prolapse	783	0.72 (0.54-0.97)	0.032	353	0.89 (0.57-1.38)	0.585
Bicuspid aortic valve	782	1.16 (0.52-2.58)	0.720	353	1.16 (0.35-3.91)	0.808
Cardiac surgical intervention						
Aortic root replacement or repair	783	2.47 (1.81-3.37)	<0.001	353	2.63 (1.65-4.19)	<0.001
Aortic valve surgery	783	2.10 (1.52-2.89)	<0.001	353	1.87 (1.15-3.04)	0.012
Mitral valve surgery	783	1.14 (0.76-1.72)	0.524	353	0.68 (0.36-1.28)	0.232
Tricuspid valve surgery	783	0.97 (0.38-2.48)	0.952	353	0.85 (0.23-3.18)	0.813

Models were adjusted for age, hypertension, diabetes mellitus, chronic kidney disease, and heart failure. n reflects the complete-case sample size for each model in the overall cohort, and GP n reflects the complete-case sample size for the genetically positive subgroup. Two-tailed *P* values less than 0.05 were considered statistically significant.

AR = aortic regurgitation; GP = genetically positive; MR = mitral regurgitation; TR = tricuspid regurgitation; other abbreviation as in Table 3.

## Results

### Study population and baseline characteristics

A total of 783 patients with confirmed MFS were included (443 men [56.6%] and 340 women [43.4%]). Mean age was similar between men and women (52.1 ± 15.2 vs 53.2 ± 15.2 years; *P* = 0.333), as was LVEF (56.3 ± 11.1% vs 57.0 ± 10.4%; *P* = 0.315). A total of 353 patients (45.1%) were genetically positive, 186/443 men (42.0%) and 167/340 women (49.1%) (*P* = 0.051). Among genetically positive patients, 142 had missense variants, 100 had truncating variants (including frameshift or nonsense mutations), and 111 had other pathogenic or likely pathogenic *FBN1* variants for which the exact mutation was not available. Among female patients, 75/340 (22.1%) had at least 1 documented pregnancy. Men had higher prevalences of hypertension (49.2% vs 38.2%; *P* = 0.002) and CKD (13.8% vs 8.8%; *P* = 0.033), whereas women had a higher prevalence of migraine (20.9% vs 10.2%; *P* < 0.001). Stroke or transient ischemic attack was numerically more common in men (9.0% vs 5.3%; *P* = 0.054), while HF (19.0% vs 15.3%; *P* = 0.184) and DM (7.2% vs 4.1%; *P* = 0.091) did not differ significantly by sex. Baseline characteristics are summarized in Table 1.

### Aneurysms and dissections

Aneurysm, dissection, and imaging findings are summarized in Table 2; absolute and BSA-indexed aortic measurements and BSA-based root thresholds by ascending aortic aneurysm status are shown in Supplemental Tables 1A and 1B, and genetically positive subgroup findings are shown in Supplemental Table 2. Men had a higher prevalence of ascending aortic aneurysm (394/442 [89.1%] vs 241/339 [71.1%]; *P* < 0.001), abdominal aortic aneurysm (27.1% vs 17.4%; *P* = 0.001), any aneurysm (91.0% vs 75.3%; *P* < 0.001), and any extra-aortic aneurysm (36.8% vs 25.9%; *P* = 0.001). Among extra-aortic territories, sex differences were most evident for iliac, femoral, and popliteal aneurysms, while other aneurysm territories did not differ significantly by sex. These aneurysm findings were directionally similar and remained significant for the major aneurysm categories in the genetically positive subgroup.

Among patients with ascending aortic aneurysm, mean aortic root/sinus and tubular ascending diameters were 42.8 ± 7.7 and 36.8 ± 7.2, with corresponding BSA-indexed diameters of 20.7 ± 4.3 and 17.8 ± 3.7. Among patients without ascending aortic aneurysm, corresponding absolute diameters were 35.2 ± 4.6 and 31.1 ± 4.4, with BSA-indexed diameters of 18.0 ± 2.4 and 16.1 ± 2.0. Mean BSA-based upper-limit root thresholds were 39.3 ± 2.5 and 37.9 ± 2.5, respectively. Dissection prevalence did not differ by sex for type A, type B, any dissection, or any extra-aortic dissection in either the overall cohort or the genetically positive subgroup.

### Arrhythmias, conduction disease, and systolic dysfunction

Arrhythmia, conduction, and systolic function findings are summarized in Table 3. In the overall cohort, men had higher prevalences of AF, AFL, right bundle branch block, sustained VT, and VF. Among patients with AF and available timing data, age at AF diagnosis was younger in men. Left bundle branch block, SVT, nonsustained VT, LVEF <40%, and age at first VF did not differ significantly by sex in the overall cohort. In the genetically positive subgroup, AF, left bundle branch block, and sustained VT were more common in men.

### Valvular disease and cardiac surgery

Valvular disease and cardiac surgery findings are summarized in Table 4. Regurgitation severity distributions did not differ significantly by sex for aortic, mitral, or tricuspid regurgitation, and bicuspid aortic valve prevalence was similar. Women had a higher prevalence of mitral valve prolapse. Aortic root replacement or repair (63.4% vs 40.3%; *P* < 0.001) and aortic valve surgery (47.2% vs 29.7%; *P* < 0.001) were more common in men, whereas mitral valve surgery, tricuspid valve surgery, and age at first surgery did not differ significantly by sex. These aortic procedural differences persisted in the genetically positive subgroup.

### Multivariable associations with male sex

Multivariable associations are summarized in Table 5 and Central Illustration, with full model outputs provided in Supplemental Tables 4A to 4F. After adjustment for age, hypertension, DM, CKD, and HF, male sex remained independently associated with ascending aortic aneurysm, abdominal aortic aneurysm, any aneurysm, and any extra-aortic aneurysm but not with type A dissection, type B dissection, any dissection, or any extra-aortic dissection. For rhythm and conduction outcomes, male sex remained associated with AF, AFL, right bundle branch block, and sustained VT. Male sex was associated with lower odds of mitral valve prolapse and higher odds of aortic root replacement or repair and aortic valve surgery, but not mitral or tricuspid valve surgery. In the genetically positive subgroup, the most consistent associations were observed for aneurysmal outcomes, AF, sustained VT, and major aortic procedures.

## Discussion

In this cohort of 783 patients with confirmed MFS, sex was associated with a distinct pattern of cardiovascular expression rather than an isolated difference in any single domain. Men had a higher burden of thoracic and extra-aortic aneurysms, more AF, AFL and sustained VT, and more aortic root and aortic valve surgery. In contrast, aortic dissection prevalence was similar between sexes, while women more frequently had mitral valve prolapse. Importantly, the major vascular findings, the excess burden of AF and sustained VT, and the procedural differences all persisted in the genetically positive subgroup, supporting that these observations are unlikely to be explained solely by diagnostic uncertainty and instead reflect meaningful variation within confirmed MFS.^11^, ^12^, ^13^, ^14^, ^15^^,^^21^^,^^22^

The vascular findings were the most consistent signal in the study. Men remained independently associated with ascending aortic aneurysm, abdominal aortic aneurysm, any aneurysm, and any extra-aortic aneurysm after adjustment for age and major clinical covariates, and these associations were preserved in the genetically positive subgroup. In a retrospective cohort where individual findings can be influenced by imaging frequency, referral patterns, and ascertainment, replication of the central vascular signal within the genetically positive subgroup strengthens confidence that the association is real and not simply a consequence of diagnostic heterogeneity. These findings also align with prior Marfan literature suggesting a heavier aortic phenotype in men, including greater aortic event burden and procedural exposure over time.^11^, ^12^, ^13^, ^14^, ^15^, ^16^ At the same time, our data reflect cumulative documented disease burden rather than onset or growth rate. Accordingly, the present study supports a greater overall aneurysmal burden in men, but it cannot determine whether that burden reflects earlier disease onset, faster enlargement, longer duration of detectable disease, or differences in surveillance.

This broader vascular phenotype extended beyond the thoracic aorta. Men had higher adjusted odds of any extra-aortic aneurysm, and unadjusted comparisons suggested that this excess was driven most clearly by iliac aneurysm. This territorial pattern argues against the extra-aortic signal being merely a vague spillover effect of more imaging or a generalized statistical artifact and instead suggests that, in at least a subset of male patients, MFS may manifest as a more diffuse arteriopathy involving branch and peripheral vessels in addition to the ascending aorta.^26^^,^^29^^,^^30^ Still, caution remains essential. Vascular imaging in this cohort was clinically driven and not standardized across all vascular beds, so the absence of documented extra-aortic disease cannot be equated with the absence of disease. For that reason, our results are better interpreted as demonstrating clinically recognized extra-aortic aneurysmal burden rather than true-territory specific prevalence. Even so, the persistence of the overall extra-aortic aneurysm association in the genetically positive subgroup argues that this is a meaningful component of phenotype and not simply noise within the medical record.^2^^,^^26^^,^^29^^,^^30^

Notably, the higher aneurysmal burden in men did not translate into a higher prevalence of dissection. Rates of type A, type B, and any dissection were similar between sexes in both the overall cohort and the genetically positive subgroup. This distinction is clinically important since it suggests that a greater burden of aneurysmal disease did not correspond to a greater documented burden of dissection in this cohort. Contemporary Marfan care emphasizes serial imaging, medical therapy, and prophylactic surgery before catastrophic aortic events occur, and the higher prevalence of aortic root and aortic valve intervention in men may reflect one pathway through which greater aneurysmal disease is recognized and treated before dissection develops.^2^^,^^12^^,^^13^^,^^15^ It is also likely that dissection risk depends on factors not captured here, including absolute aortic dimensions, rate of growth, treatment exposure, pregnancy-related stressors, and genotype-specific severity.^2^^,^^6^^,^^9^^,^^17^^,^^21^^,^^22^ Taken together, these findings suggest that the relationship between aneurysmal disease and dissection is likely modified by additional clinical and biologic factors not captured by prevalence alone.

The rhythm findings also deserve greater emphasis than they have typically received in Marfan cohorts. Men had a higher burden of AF and AFL, and AF remained independently associated with male sex in both the overall and genetically positive cohorts. Men were also diagnosed with AF at a younger age among those with available timing data. Beyond atrial arrhythmias, sustained VT was more common in men, remained independently associated with male sex after adjustment, and persisted in the genetically positive subgroup with a larger effect estimate. VF was likewise more frequent in men in the overall cohort, although the smaller number of subgroup events warrants caution in how heavily that finding is weighted. Importantly, these differences were not accompanied by a significant sex difference in EF < 40%, arguing against the excess arrhythmic burden in men being explained simply by more overt systolic dysfunction. Prior work has shown that patients with MFS can have myocardial involvement, ventricular ectopy, and clinically significant arrhythmias, with adverse rhythm phenotypes often clustering in those with valvular abnormalities, ventricular remodeling, or prior cardiac surgery.^19^^,^^23^, ^24^, ^25^, ^26^, ^27^^,^^31^ Mitral annular disjunction has also been associated with adverse cardiovascular outcomes in MFS and may represent one structural substrate contributing to arrhythmic vulnerability in selected patients.^23^ Although the present study cannot distinguish whether the higher burden of atrial and ventricular arrhythmias in men reflects intrinsic sex-related myocardial differences, greater cumulative aortic and surgical disease, or differences in rhythm surveillance, the persistence of AF and sustained VT in the genetically positive subgroup supports that this is a clinically meaningful rhythm phenotype rather than an incidental finding.

The valvular and procedural findings add important granularity to the sex-based phenotype. Women had a higher prevalence of mitral valve prolapse, and male sex was associated with lower adjusted odds of prolapse, yet this did not translate into more moderate-severe mitral regurgitation or greater mitral valve surgery. This suggests that the excess prolapse signal in women reflects a difference in structural phenotype rather than a clearer difference in downstream mitral severity within this cohort. By contrast, the procedural signal in men was concentrated in aortic root replacement or repair and aortic valve surgery, not in mitral or tricuspid operations, a pattern that closely parallels the vascular findings and suggests that sex differences in intervention are driven predominantly by aortic phenotype rather than diffuse excess surgical disease across all valves. The nonsignificant trend toward more moderate-severe aortic regurgitation in men is directionally consistent with this interpretation. Notably, despite the markedly higher lifetime prevalence of aortic procedures in men, age at first cardiac surgery did not differ significantly by sex, suggesting that the operative difference is better understood as greater cumulative procedural burden rather than clearly earlier surgical timing.^2^^,^^3^^,^^23^^,^^30^

The genetically positive subgroup substantially strengthens the interpretation of the study and helps distinguish the most reliable signals from the more fragile ones. Within pathogenic or likely pathogenic *FBN1*-positive disease, men continued to show greater odds of ascending aortic aneurysm, abdominal aortic aneurysm, any aneurysm, any extra-aortic aneurysm, AF, sustained VT, aortic root replacement or repair, and aortic valve surgery, while dissection remained similar.^32^ This demonstrates that the central vascular, rhythm, and aortic procedural findings survive restriction to patients with stronger molecular confirmation of diagnosis, which is especially important in a retrospective study spanning multiple sites and modes of ascertainment.^11^^,^^14^^,^^21^^,^^22^ At the same time, the subgroup analysis was informative not only for what remained significant but also for what did not. AFL, right bundle branch block, and mitral valve prolapse were less robust in the genetically positive subgroup, suggesting that these associations may be weaker, more sensitive to sample size, or more contingent on broader phenotypic heterogeneity. In that sense, the genetic-only analysis helps prioritize the findings most likely to reflect durable sex-linked differences in MFS: aneurysmal burden across vascular beds, AF, sustained VT, and aortic intervention.

Clinically, these findings support a sex-aware interpretation of cardiovascular phenotype in MFS, but not a sex-specific management framework based on retrospective prevalence data alone.^2^^,^^3^ The more measured implication is that men with MFS may warrant especially careful attention to diffuse aneurysmal burden and clinically meaningful rhythm disease, whereas women may more often manifest a prolapse-predominant valvular phenotype within an otherwise substantial cardiovascular disease burden. Future studies should build on this by integrating standardized vascular imaging, serial aortic dimensions, medication exposure, genotype subclassification, pregnancy history, operative indications, and structured rhythm surveillance. Only with that level of longitudinal phenotyping will it be possible to determine whether the sex differences observed here reflect earlier disease onset, faster progression, distinct mechanisms of tissue vulnerability, or different pathways to intervention and adverse outcomes.^2^^,^^6^^,^^17^^,^^19^^,^^23^, ^24^, ^25^, ^26^^,^^29^

### Study Limitations

This study has several limitations. First, its retrospective design depends on the completeness and accuracy of clinical documentation and remains subject to selection and detection bias, despite manual confirmation of Marfan diagnoses and vascular outcomes. Second, vascular imaging was obtained for clinical rather than research purposes and was not standardized across patients, sites, or vascular territories; accordingly, extra-aortic disease may have been underrecognized, and observed sex differences could have been influenced by differences in imaging intensity or anatomic coverage. In addition, classification of abdominal aortic and extra-aortic aneurysms relied on radiology-report terminology rather than uniform retrospective diameter thresholds across all vascular beds, which may have introduced ascertainment heterogeneity between arterial territories. Third, outcomes were analyzed as ever documented in the medical record, so our findings reflect cumulative disease burden rather than temporal sequence. We therefore could not determine age at onset, aneurysm growth rates, time to dissection, or the extent to which prophylactic intervention may have influenced downstream dissection patterns. This approach may also partly explain why arrhythmia prevalence appeared higher than in some prior cohorts, particularly in a tertiary referral population with long follow-up and substantial prior surgical exposure. Fourth, although a genetically positive sensitivity analysis was performed, molecular confirmation and detailed genotype data were not uniformly available across the full cohort, limiting more granular genotype-phenotype analyses. Fifth, although multivariable models adjusted for prespecified clinical covariates, residual confounding is likely, including from body size, medication exposure and adherence, hemodynamic factors, pregnancy-related history, and other care-related differences. Finally, structural substrates potentially relevant to sex differences in arrhythmia and valvular phenotype, including mitral annular disjunction and other imaging-derived myocardial features, were not systematically assessed.

**Central Illustration:**
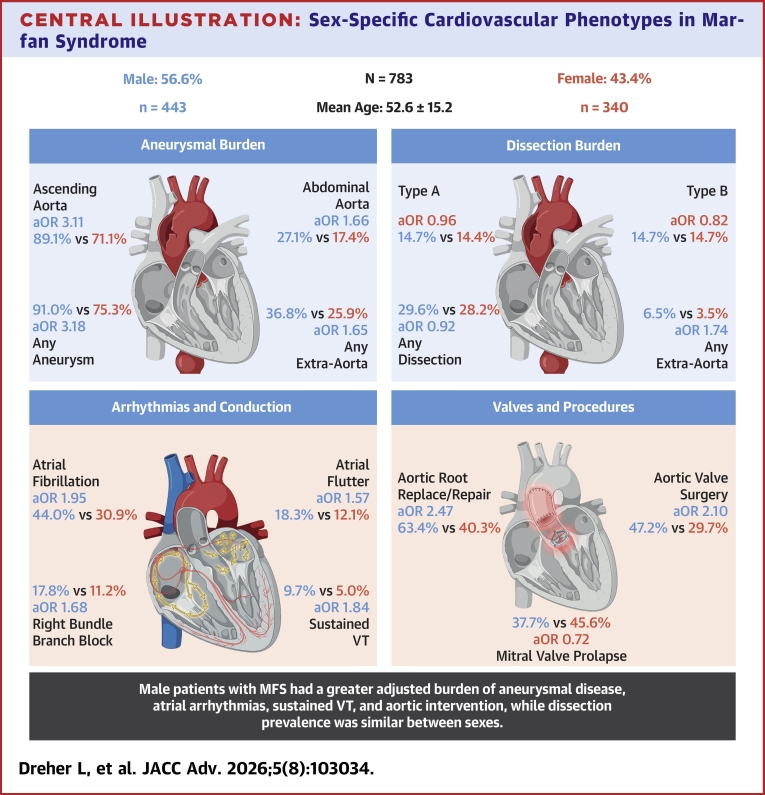
**Sex-Specific Cardiovascular Phenotypes in Marfan Syndrome** Among 783 adults with confirmed Marfan syndrome, male patients demonstrated higher adjusted odds of aneurysmal disease, atrial arrhythmias, sustained ventricular tachycardia, and major aortic intervention. In contrast, dissection prevalence was similar between sexes, and mitral valve prolapse was more frequent among female patients. Adjusted ORs depict the association between male sex and each cardiovascular phenotype; teal adjusted ORs indicate higher adjusted odds among male patients, whereas red adjusted ORs indicate lower adjusted odds among male patients. Blue and red percentages represent unadjusted prevalence among male and female patients, respectively. Aneurysmal, atrial fibrillation, and sustained ventricular tachycardia associations remained significant in the genetically positive subgroup. Thus, greater aneurysmal burden among men did not translate into higher adjusted dissection prevalence; women more frequently had mitral valve prolapse, whereas men more frequently underwent major aortic procedures. Created in BioRender, Dreher L. (2026), https://BioRender.com/kb9bqvd. aOR = adjusted OR; MFS = Marfan syndrome; VT = ventricular tachycardia

## Conclusions

In this multicenter cohort of 783 adults with confirmed MFS, men had a greater burden of thoracic and extra-aortic aneurysmal disease, more AF, AFL, and sustained VT, and more aortic intervention, whereas aortic dissection prevalence was similar between sexes. Women more frequently had mitral valve prolapse. Together, these findings support sex-associated differences in the vascular and cardiac expression of MFS and reinforce the need for comprehensive cardiovascular surveillance. Longitudinal studies with standardized imaging, genotype integration, and structured rhythm assessment are needed to clarify how these differences influence disease progression and long-term outcomes.

Perspectives **COMPETENCY IN MEDICAL KNOWLEDGE:** In adults with confirmed MFS, men demonstrated a greater burden of thoracic and extra-aortic aneurysmal disease, AF, AFL, sustained VT, and aortic intervention, whereas women more frequently had mitral valve prolapse. These findings support comprehensive cardiovascular surveillance in both sexes while recognizing that disease expression may differ by sex across vascular, rhythm, and valvular domains.

**TRANSLATIONAL OUTLOOK:** Future longitudinal studies should incorporate standardized vascular imaging, genotype-specific analyses, pregnancy history, and structured rhythm surveillance to determine whether these sex-associated differences reflect earlier disease onset, faster progression, or distinct pathways to intervention and long-term outcomes.

## Funding support and author disclosures

The authors have reported that they have no relationships relevant to the contents of this paper to disclose.
