# Supplementary material for: Sex-Specific Cardiovascular Phenotypes in Marfan Syndrome
Source: JACC Adv. 2026 Jul 20;5(8):103034. doi: 10.1016/j.jacadv.2026.103034 (PMC13393658; doi:10.1016/j.jacadv.2026.103034)
Supplement: Supplementary_Tables_1-4 [file mmc1.pdf]

Supplementary Table 1. Absolute and BSA-indexed aortic measurements and BSA-based root thresholds stratified by ascending aortic aneurysm status and sex

Supplementary Table 1A. Patients with ascending aortic aneurysm

Measurement	Overall	Male	Female	p value
Aortic root/sinus diameter, mm	42.8 ± 7.7 (n = 497)	43.5 ± 8.1 (n = 305)	41.6 ± 6.9 (n = 192)	0.010
Maximum root/sinus diameter indexed to BSA, mm/m^2	20.7 ± 4.3 (n = 451)	20.0 ± 4.2 (n = 276)	21.7 ± 4.2 (n = 175)	< 0.001
Aortic tubular ascending diameter, mm	36.8 ± 7.2 (n = 553)	37.9 ± 7.2 (n = 338)	35.2 ± 6.8 (n = 215)	< 0.001
Maximum tubular ascending diameter indexed to BSA, mm/m^2	17.8 ± 3.7 (n = 502)	17.5 ± 3.7 (n = 306)	18.3 ± 3.6 (n = 196)	0.029
BSA-based upper-limit root threshold, mm	39.3 ± 2.5 (n = 512)	40.7 ± 1.8 (n = 315)	37.0 ± 1.7 (n = 197)	< 0.001

Supplementary Table 1B. Patients without ascending aortic aneurysm

Measurement	Overall	Male	Female	p value
Aortic root/sinus diameter, mm	35.2 ± 4.6 (n = 95)	36.5 ± 4.7 (n = 31)	34.6 ± 4.5 (n = 64)	0.060
Maximum root/sinus diameter indexed to BSA, mm/m²	18.0 ± 2.4 (n = 79)	17.5 ± 2.6 (n = 25)	18.2 ± 2.3 (n = 54)	0.216
Aortic tubular ascending diameter, mm	31.1 ± 4.4 (n = 101)	32.2 ± 4.5 (n = 33)	30.6 ± 4.4 (n = 68)	0.085
Maximum tubular ascending diameter indexed to BSA, mm/m²	16.1 ± 2.0 (n = 86)	15.4 ± 1.7 (n = 28)	16.5 ± 2.1 (n = 58)	0.024
BSA-based upper-limit root threshold, mm	37.9 ± 2.5 (n = 88)	40.2 ± 2.0 (n = 28)	36.8 ± 1.9 (n = 60)	< 0.001

Values are presented as mean ± SD, with the number of patients with available measurements shown in parentheses. P-values reflect sex-based comparisons. BSA indicates body surface area.

Supplementary Table 2. Sensitivity analysis restricted to genetically confirmed patients with positive genetic testing (n = 353)

**Variable**	**Overall Cohort (n = 353)**	**Male (n = 186)**	**Female (n = 167)**	**p value**
**Age, years (mean ± SD)**	50.10 ± 14.04	50.01 ± 13.83	50.19 ± 14.31	> 0.900
**Follow up time, years (mean ± SD)**	12.37 ± 6.94	12.16 ± 6.85	12.60 ± 7.05	0.554
**Left Ventricular EF (%), mean ± SD**	56.23 ± 11.27	55.98 ± 11.41	56.51 ± 11.13	0.658
**Hypertension, n (%)**	155 (43.9%)	89 (47.8%)	66 (39.5%)	0.133
**Chronic Kidney Disease, n (%)**	37 (10.5%)	24 (12.9%)	13 (7.8%)	0.163
**Migraines, n (%)**	60 (17.0%)	17 (9.1%)	43 (25.7%)	< 0.001
**Stroke or TIA, n (%)**	26 (7.4%)	19 (10.2%)	7 (4.2%)	0.040
**Heart Failure, n (%)**	70 (19.8%)	39 (21.0%)	31 (18.6%)	0.595
**Diabetes Mellitus, n (%)**	15 (4.2%)	11 (5.9%)	4 (2.4%)	0.119
**Aneurysms**				
Ascending Aortic, n (%)	302 (85.8%)	175 (94.1%)	127 (76.5%)	< 0.001
Abdominal Aortic, n (%)	83 (23.5%)	53 (28.5%)	30 (18.0%)	0.024
Any Aneurysm, n (%)	308 (87.3%)	176 (94.6%)	132 (79.0%)	< 0.001
Any Extra-Aortic Aneurysm, n (%)	118 (33.4%)	72 (38.7%)	46 (27.5%)	0.032
**Dissections**				
Type A Dissection, n (%)	55 (15.6%)	30 (16.1%)	25 (15.0%)	0.771
Type B Dissection, n (%)	43 (12.2%)	20 (10.8%)	23 (13.8%)	0.418
Any Dissection, n (%)	103 (29.2%)	53 (28.5%)	50 (29.9%)	0.815
Any Extra-Aortic Dissection, n (%)	23 (6.5%)	16 (8.6%)	7 (4.2%)	0.130

**Imaging (CT / MR)**				
Head and Neck, n (%)	190 (53.8%)	103 (55.4%)	87 (52.1%)	0.593
Chest, n (%)	313 (88.7%)	165 (88.7%)	148 (88.6%)	> 0.900
Abdomen and Pelvis, n (%)	287 (81.3%)	150 (80.6%)	137 (82.0%)	0.785
**Arrhythmias and systolic dysfunction**				
Atrial Fibrillation, n (%)	118 (33.4%)	73 (39.2%)	45 (26.9%)	0.018
Age at Atrial Fibrillation Diagnosis, years (mean ± SD)	48.25 ± 12.13 (112)	46.98 ± 11.87 (68)	50.21 ± 12.40 (44)	0.175
Atrial Flutter, n (%)	54 (15.3%)	33 (17.7%)	21 (12.6%)	0.186
Right Bundle Branch Block, n (%)	66 (18.7%)	42 (22.6%)	24 (14.4%)	0.056
Left Bundle Branch Block, n (%)	26 (7.4%)	19 (10.2%)	7 (4.2%)	0.040
Supraventricular Tachycardia, n (%)	69 (19.5%)	31 (16.7%)	38 (22.8%)	0.179
Nonsustained VT, n (%)	47 (13.3%)	28 (15.1%)	19 (11.4%)	0.348
Sustained VT, n (%)	28 (7.9%)	21 (11.3%)	7 (4.2%)	0.017
VF, n (%)	18 (5.1%)	13 (7.0%)	5 (3.0%)	0.096
Age at First Ventricular Fibrillation, years (mean ± SD)	46.18 ± 12.92 (15)	48.70 ± 12.93 (12)	36.08 ± 7.68 (3)	0.078
EF < 40%, n (%)	29 (8.2%)	16 (8.6%)	13 (7.8%)	0.847
**Valvular disease**				
Aortic Valve Regurgitation				0.413
Mild, n (%)	47 (13.3%)	24 (12.9%)	23 (13.8%)	
Moderate, n (%)	18 (5.1%)	9 (4.8%)	9 (5.4%)	
Severe, n (%)	44 (12.5%)	28 (15.1%)	16 (9.6%)	

Bicuspid Aortic Valve, n (%)	11 (3.1%)	6 (3.2%)	5 (3.0%)	> 0.900
Mitral Valve Regurgitation				0.351
Mild, n (%)	103 (29.2%)	57 (30.6%)	46 (27.5%)	
Moderate, n (%)	34 (9.6%)	14 (7.5%)	20 (12.0%)	
Severe, n (%)	42 (11.9%)	21 (11.3%)	21 (12.6%)	
Mitral Valve Prolapse, n (%)	148 (41.9%)	74 (39.8%)	74 (44.3%)	0.450
Tricuspid Valve Regurgitation				0.273
Mild, n (%)	103 (29.2%)	58 (31.2%)	45 (26.9%)	
Moderate, n (%)	49 (13.9%)	22 (11.8%)	27 (16.2%)	
Severe, n (%)	6 (1.7%)	2 (1.1%)	4 (2.4%)	
**Cardiac surgery**				
Aortic Root Replacement or Repair, n (%)	186 (52.7%)	119 (64.0%)	67 (40.1%)	< 0.001
Aortic Valve Surgery, n (%)	124 (35.1%)	78 (41.9%)	46 (27.5%)	0.005
Mitral Valve Surgery, n (%)	56 (15.9%)	25 (13.4%)	31 (18.6%)	0.193
Tricuspid Valve Surgery, n (%)	10 (2.8%)	5 (2.7%)	5 (3.0%)	> 0.900
Age at First Surgery, years (mean ± SD)	35.93 ± 13.11 (204)	35.83 ± 13.41 (125)	36.08 ± 12.71 (79)	0.894

Values are n (%) unless otherwise specified; continuous variables are mean ± SD. Outcomes are defined as a history ever documented during the study period and analyzed as binary variables. P-values reflect sex-based comparisons using χ^2 tests (or Fisher's exact test when expected cell counts were small) for categorical variables and parametric or nonparametric tests as appropriate for continuous variables.

Supplementary Table 3. Univariate associations with male sex (Male vs Female)

Predictor	n	OR	95% CI	p value
**Genetically Positive**	783	0.750	0.564–0.996	0.047
**Age (continuous)**	783	0.995	0.986–1.005	0.333
**Left Ventricular EF (continuous)**	777	0.993	0.980–1.007	0.319
**Hypertension**	783	1.565	1.174–2.086	0.002
**Chronic Kidney Disease**	783	1.650	1.040–2.619	0.034
**Migraine**	783	0.428	0.286–0.642	< 0.001
**Stroke or TIA**	783	1.776	0.999–3.156	0.050
**Heart Failure**	783	1.296	0.887–1.893	0.180
**Diabetes Mellitus**	783	1.813	0.952–3.454	0.070
**Ascending Aortic Aneurysm**	781	3.338	2.281–4.884	< 0.001
**Abdominal Aortic Aneurysm**	783	1.769	1.247–2.512	0.001
**Any Aneurysm**	783	3.306	2.199–4.970	< 0.001
**Any Extra-Aortic Aneurysm**	783	1.667	1.223–2.273	0.001
**Type A Dissection**	783	1.021	0.684–1.525	0.918
**Type B Dissection**	783	0.997	0.669–1.487	0.990
**Any Dissection**	783	1.067	0.781–1.458	0.683
**Any Extra-Aortic Dissection**	783	1.915	0.962–3.810	0.064
**Atrial Fibrillation**	783	1.760	1.308–2.368	< 0.001
**Atrial Flutter**	783	1.632	1.088–2.448	0.018
**Right Bundle Branch Block**	783	1.725	1.138–2.614	0.010
**Left Bundle Branch Block**	783	1.582	0.882–2.838	0.124

**Supraventricular Tachycardia**	783	0.752	0.519–1.089	0.131
**Nonsustained Ventricular Tachycardia**	783	1.297	0.835–2.017	0.247
**Sustained Ventricular Tachycardia**	783	2.043	1.143–3.649	0.016
**Ventricular Fibrillation**	783	2.173	1.073–4.401	0.031
**Moderate–Severe Aortic Regurgitation**	783	1.555	1.069–2.264	0.020
**Moderate–Severe Tricuspid Regurgitation**	783	0.771	0.528–1.127	0.204
**Moderate–Severe Mitral Regurgitation**	783	0.938	0.670–1.313	0.731
**Mitral Valve Prolapse**	783	0.722	0.542–0.962	0.026
**Bicuspid Aortic Valve**	782	1.051	0.476–2.318	0.903
**Aortic Root Replacement or Repair**	783	2.570	1.922–3.436	< 0.001
**Aortic Valve Surgery**	783	2.114	1.569–2.848	< 0.001
**Mitral Valve Surgery**	783	1.155	0.784–1.702	0.467
**Tricuspid Valve Surgery**	783	1.155	0.467–2.859	0.755

Values shown are odds ratios (ORs) with 95% confidence intervals (CI) from univariate logistic regression models comparing male vs female. Two-tailed p-values <0.05 were considered statistically significant.

Supplementary Table 4. Multivariable logistic regression models for prespecified outcomes (full model outputs)

S4A. Cerebrovascular

Outcome	Predictor	OR (95% CI)	p value	GP OR (95% CI)	GP p value
**Stroke/TIA**	Male (vs Female)	1.51 (0.83–2.76)	0.178	2.58 (1.01–6.61)	0.049
	Age (years)	0.99 (0.97–1.01)	0.234	1.02 (0.98–1.05)	0.314
	Hypertension	2.98 (1.56–5.71)	< 0.001	3.08 (1.12–8.46)	0.029
	Diabetes Mellitus	1.14 (0.43–3.03)	0.800	0.30 (0.03–2.62)	0.276
	Chronic Kidney Disease	1.65 (0.82–3.31)	0.162	1.55 (0.53–4.57)	0.428
	Heart Failure	4.15 (2.29–7.53)	< 0.001	4.04 (1.64–9.96)	0.002

Values are presented as odds ratios (ORs) with 95% confidence intervals (CIs) from multivariable logistic regression models in the overall cohort and genetically positive (GP) subgroup. Models were adjusted for age, hypertension, diabetes mellitus, chronic kidney disease, and heart failure. Model sample sizes were 783 for the overall cohort and 353 for the GP subgroup for Stroke or TIA. P values are two-tailed.

S4B. Aneurysms

Outcome	Predictor	OR (95% CI)	p value	GP OR (95% CI)	GP p value
**Ascending Aortic Aneurysm**	Male (vs Female)	3.11 (2.10–4.60)	< 0.001	4.69 (2.28–9.66)	< 0.001
	Age (years)	1.00 (0.99–1.02)	0.684	1.00 (0.98–1.03)	0.773
	Hypertension	1.81 (1.18–2.77)	0.007	1.61 (0.78–3.33)	0.201
	Diabetes Mellitus	0.88 (0.34–2.30)	0.796	0.83 (0.09–7.71)	0.867
	Chronic Kidney Disease	1.53 (0.66–3.58)	0.324	3.48 (0.42–28.62)	0.246
	Heart Failure	4.62 (1.95–10.96)	< 0.001	5.14 (1.17–22.52)	0.030
**Abdominal Aortic Aneurysm**	Male (vs Female)	1.66 (1.14–2.42)	0.008	1.80 (1.03–3.14)	0.039
	Age (years)	1.02 (1.01–1.04)	< 0.001	1.04 (1.02–1.06)	< 0.001
	Hypertension	2.61 (1.78–3.83)	< 0.001	2.95 (1.65–5.28)	< 0.001
	Diabetes Mellitus	0.76 (0.38–1.54)	0.445	0.77 (0.24–2.48)	0.662
	Chronic Kidney Disease	2.04 (1.24–3.36)	0.005	2.17 (0.99–4.77)	0.055
	Heart Failure	1.90 (1.23–2.94)	0.004	1.39 (0.73–2.64)	0.321
**Any Aneurysm**	Male (vs Female)	3.18 (2.09–4.85)	< 0.001	4.53 (2.13–9.63)	< 0.001
	Age (years)	1.02 (1.00–1.03)	0.030	1.02 (0.99–1.04)	0.236
	Hypertension	1.85 (1.16–2.94)	0.010	1.87 (0.86–4.09)	0.115
	Diabetes Mellitus	0.61 (0.23–1.60)	0.313	0.66 (0.07–6.19)	0.718
	Chronic Kidney Disease	1.74 (0.66–4.61)	0.265	2.99 (0.37–24.27)	0.305
	Heart Failure	3.58 (1.50–8.53)	0.004	2.82 (0.81–9.87)	0.105

**Any Extra-Aortic Aneurysm**	Male (vs Female)	1.65 (1.18–2.29)	0.003	1.72 (1.06–2.80)	0.028
	Age (years)	1.03 (1.02–1.04)	< 0.001	1.03 (1.01–1.05)	< 0.001
	Hypertension	2.22 (1.59–3.10)	< 0.001	2.68 (1.62–4.42)	< 0.001
	Diabetes Mellitus	0.44 (0.22–0.87)	0.019	0.28 (0.08–0.95)	0.042
	Chronic Kidney Disease	1.28 (0.78–2.09)	0.333	0.96 (0.44–2.11)	0.924
	Heart Failure	1.72 (1.14–2.60)	0.010	1.33 (0.73–2.43)	0.357

Values are presented as odds ratios (ORs) with 95% confidence intervals (CIs) from multivariable logistic regression models in the overall cohort and genetically positive (GP) subgroup. Models were adjusted for age, hypertension, diabetes mellitus, chronic kidney disease, and heart failure. For the outcomes shown here, model sample sizes were 781 in the overall cohort and 352 in the GP subgroup for ascending aortic aneurysm, and 783 in the overall cohort and 353 in the GP subgroup for abdominal aortic aneurysm, any aneurysm, and any extra-aortic aneurysm.

S4C. Dissections

Outcome	Predictor	OR (95% CI)	p value	GP OR (95% CI)	GP p value
**Type A Dissection**	Male (vs Female)	0.96 (0.63–1.45)	0.845	1.01 (0.55–1.86)	0.983
	Age (years)	1.01 (1.00–1.03)	0.102	1.02 (1.00–1.04)	0.097
	Hypertension	2.72 (1.75–4.25)	< 0.001	3.49 (1.76–6.89)	< 0.001
	Diabetes Mellitus	0.15 (0.04–0.64)	0.011	0.16 (0.02–1.27)	0.082
	Chronic Kidney Disease	1.03 (0.55–1.91)	0.929	1.76 (0.73–4.24)	0.210
	Heart Failure	1.58 (0.95–2.62)	0.078	1.52 (0.74–3.10)	0.252
**Type B Dissection**	Male (vs Female)	0.82 (0.54–1.25)	0.356	0.62 (0.31–1.21)	0.158
	Age (years)	1.00 (0.98–1.01)	0.897	0.99 (0.97–1.02)	0.576
	Hypertension	3.42 (2.14–5.46)	< 0.001	3.27 (1.54–6.96)	0.002
	Diabetes Mellitus	0.97 (0.44–2.12)	0.932	1.13 (0.28–4.53)	0.866
	Chronic Kidney Disease	2.07 (1.20–3.58)	0.009	2.57 (1.04–6.33)	0.040
	Heart Failure	1.87 (1.15–3.04)	0.012	1.38 (0.64–2.98)	0.411
**Any Dissection**	Male (vs Female)	0.92 (0.65–1.29)	0.624	0.77 (0.46–1.28)	0.310
	Age (years)	1.01 (1.00–1.03)	0.034	1.02 (1.00–1.04)	0.052
	Hypertension	3.54 (2.49–5.04)	< 0.001	4.75 (2.75–8.23)	< 0.001
	Diabetes Mellitus	0.45 (0.22–0.93)	0.030	0.64 (0.20–2.09)	0.462
	Chronic Kidney Disease	1.88 (1.15–3.10)	0.013	2.76 (1.25–6.11)	0.012
	Heart Failure	1.79 (1.17–2.73)	0.007	1.21 (0.65–2.26)	0.550

**Any Extra-Aortic Dissection**	Male (vs Female)	1.74 (0.87–3.52)	0.120	1.94 (0.75–5.04)	0.172
	Age (years)	1.02 (0.99–1.04)	0.142	1.03 (0.99–1.06)	0.138
	Hypertension	2.86 (1.36–6.03)	0.006	3.72 (1.26–11.00)	0.017
	Diabetes Mellitus	1.02 (0.33–3.10)	0.979	3.24 (0.86–12.24)	0.083
	Chronic Kidney Disease	1.18 (0.49–2.84)	0.707	1.27 (0.36–4.45)	0.708
	Heart Failure	1.10 (0.50–2.44)	0.806	0.44 (0.13–1.49)	0.187

Values are presented as odds ratios (ORs) with 95% confidence intervals (CIs) from multivariable logistic regression models in the overall cohort and genetically positive (GP) subgroup, adjusted for age, hypertension, diabetes mellitus, chronic kidney disease, and heart failure. Model sample sizes were 783/353 for type A dissection, type B dissection, any dissection, and any extra-aortic dissection (overall cohort/GP subgroup).

S4D. Arrhythmias

Outcome	Predictor	OR (95% CI)	p value	GP OR (95% CI)	GP p value
**Atrial Fibrillation**	Male (vs Female)	1.95 (1.39–2.75)	< 0.001	1.94 (1.15–3.29)	0.014
	Age (years)	1.06 (1.04–1.07)	< 0.001	1.06 (1.04–1.09)	< 0.001
	Hypertension	1.20 (0.85–1.70)	0.300	1.40 (0.82–2.41)	0.221
	Diabetes Mellitus	0.88 (0.44–1.78)	0.724	0.68 (0.19–2.41)	0.554
	Chronic Kidney Disease	1.92 (1.12–3.29)	0.019	2.10 (0.92–4.81)	0.078
	Heart Failure	5.02 (3.16–7.97)	< 0.001	4.37 (2.30–8.31)	< 0.001
**Atrial Flutter**	Male (vs Female)	1.57 (1.01–2.44)	0.047	1.42 (0.74–2.70)	0.292
	Age (years)	1.04 (1.03–1.06)	< 0.001	1.04 (1.02–1.07)	0.001
	Hypertension	1.65 (1.05–2.60)	0.030	2.26 (1.13–4.51)	0.021
	Diabetes Mellitus	1.10 (0.53–2.31)	0.793	0.45 (0.11–1.88)	0.276
	Chronic Kidney Disease	2.65 (1.56–4.51)	< 0.001	3.44 (1.52–7.81)	0.003
	Heart Failure	2.55 (1.58–4.10)	< 0.001	1.70 (0.83–3.48)	0.149
**Right Bundle Branch Block**	Male (vs Female)	1.68 (1.10–2.57)	0.017	1.73 (0.99–3.03)	0.056
	Age (years)	0.97 (0.96–0.99)	< 0.001	0.98 (0.96–1.00)	0.059
	Hypertension	1.33 (0.87–2.04)	0.192	1.54 (0.86–2.78)	0.151
	Diabetes Mellitus	0.83 (0.31–2.23)	0.709	0.61 (0.13–2.90)	0.530
	Chronic Kidney Disease	0.63 (0.29–1.35)	0.233	0.87 (0.33–2.32)	0.785
	Heart Failure	1.15 (0.66–2.00)	0.617	0.89 (0.42–1.85)	0.745

**Left Bundle Branch Block**	Male (vs Female)	1.50 (0.83–2.72)	0.178	2.43 (0.98–6.02)	0.055
	Age (years)	0.99 (0.97–1.01)	0.498	1.00 (0.97–1.03)	0.997
	Hypertension	1.53 (0.84–2.77)	0.165	1.88 (0.77–4.59)	0.165
	Diabetes Mellitus	1.19 (0.40–3.60)	0.752	2.31 (0.56–9.62)	0.249
	Chronic Kidney Disease	0.82 (0.32–2.09)	0.676	0.45 (0.10–2.13)	0.314
	Heart Failure	1.05 (0.50–2.22)	0.901	1.38 (0.52–3.65)	0.520
**Supraventricular Tachycardia**	Male (vs Female)	0.73 (0.50–1.07)	0.104	0.65 (0.38–1.12)	0.119
	Age (years)	1.01 (1.00–1.02)	0.119	1.00 (0.98–1.02)	0.866
	Hypertension	1.00 (0.67–1.49)	0.984	1.39 (0.78–2.47)	0.270
	Diabetes Mellitus	0.67 (0.29–1.58)	0.362	0.51 (0.11–2.42)	0.398
	Chronic Kidney Disease	1.53 (0.88–2.68)	0.131	1.53 (0.66–3.55)	0.323
	Heart Failure	1.67 (1.04–2.67)	0.034	1.23 (0.63–2.42)	0.542
**Nonsustained VT**	Male (vs Female)	1.17 (0.73–1.86)	0.509	1.37 (0.71–2.63)	0.350
	Age (years)	1.02 (1.00–1.03)	0.033	1.02 (1.00–1.05)	0.084
	Hypertension	1.11 (0.68–1.81)	0.674	1.36 (0.68–2.72)	0.390
	Diabetes Mellitus	2.03 (0.96–4.26)	0.063	0.46 (0.09–2.35)	0.353
	Chronic Kidney Disease	1.58 (0.87–2.85)	0.132	0.95 (0.37–2.45)	0.910
	Heart Failure	3.71 (2.26–6.07)	< 0.001	3.80 (1.88–7.69)	< 0.001
**Sustained VT**	Male (vs Female)	1.84 (1.01–3.35)	0.045	2.80 (1.12–7.04)	0.028
	Age (years)	1.00 (0.98–1.02)	0.808	1.02 (0.99–1.05)	0.253

	Hypertension	1.04 (0.57–1.88)	0.900	1.08 (0.44–2.65)	0.875
	Diabetes Mellitus	2.36 (1.01–5.53)	0.048	1.44 (0.32–6.42)	0.635
	Chronic Kidney Disease	1.35 (0.66–2.77)	0.412	1.42 (0.50–4.07)	0.511
	Heart Failure	3.60 (1.99–6.50)	< 0.001	5.50 (2.30–13.15)	< 0.001

Values are presented as odds ratios (ORs) with 95% confidence intervals (CIs) from multivariable logistic regression models in the overall cohort and genetically positive (GP) subgroup, adjusted for age, hypertension, diabetes mellitus, chronic kidney disease, and heart failure. Model sample sizes were 783/353 for atrial fibrillation, atrial flutter, right bundle branch block, left bundle branch block, supraventricular tachycardia, nonsustained ventricular tachycardia, and sustained ventricular tachycardia (overall cohort/GP subgroup).

S4E. Valvular Disease

Outcome	Predictor	OR (95% CI)	p value	GP OR (95% CI)	GP p value
**Moderate–Severe AR**	Male (vs Female)	1.47 (0.99–2.18)	0.057	1.38 (0.77–2.49)	0.278
	Age (years)	1.01 (1.00–1.03)	0.076	0.99 (0.97–1.01)	0.263
	Hypertension	2.37 (1.58–3.57)	< 0.001	2.81 (1.50–5.25)	0.001
	Diabetes Mellitus	0.58 (0.26–1.30)	0.187	0.18 (0.02–1.43)	0.104
	Chronic Kidney Disease	0.99 (0.57–1.73)	0.976	1.00 (0.40–2.47)	0.992
	Heart Failure	2.86 (1.84–4.46)	< 0.001	2.74 (1.41–5.31)	0.003
**Moderate–Severe TR**	Male (vs Female)	0.71 (0.47–1.06)	0.093	0.59 (0.32–1.09)	0.093
	Age (years)	1.03 (1.01–1.04)	< 0.001	1.04 (1.01–1.06)	0.003
	Hypertension	1.12 (0.73–1.72)	0.598	1.52 (0.79–2.94)	0.209
	Diabetes Mellitus	0.91 (0.42–1.99)	0.814	0.69 (0.17–2.83)	0.608
	Chronic Kidney Disease	1.42 (0.81–2.48)	0.219	0.98 (0.39–2.46)	0.969
	Heart Failure	3.15 (1.99–4.98)	< 0.001	2.99 (1.51–5.92)	0.002
**Moderate–Severe MR**	Male (vs Female)	0.90 (0.63–1.30)	0.579	0.69 (0.39–1.20)	0.188
	Age (years)	1.02 (1.00–1.03)	0.011	1.02 (0.99–1.04)	0.177
	Hypertension	0.77 (0.52–1.14)	0.186	0.43 (0.23–0.82)	0.011
	Diabetes Mellitus	0.36 (0.14–0.87)	0.024	0.65 (0.15–2.89)	0.573
	Chronic Kidney Disease	1.19 (0.69–2.07)	0.531	0.70 (0.28–1.74)	0.444
	Heart Failure	6.23 (4.05–9.59)	< 0.001	8.44 (4.32–16.51)	< 0.001

**Mitral Valve Prolapse**	Male (vs Female)	0.72 (0.54–0.97)	0.032	0.89 (0.57–1.38)	0.585
	Age (years)	1.00 (0.99–1.01)	0.620	1.01 (0.99–1.03)	0.327
	Hypertension	0.66 (0.48–0.90)	0.009	0.43 (0.27–0.71)	< 0.001
	Diabetes Mellitus	0.93 (0.49–1.76)	0.817	0.33 (0.09–1.29)	0.112
	Chronic Kidney Disease	1.10 (0.68–1.79)	0.691	1.05 (0.49–2.25)	0.907
	Heart Failure	2.35 (1.58–3.51)	< 0.001	2.67 (1.48–4.81)	0.001
**Bicuspid Aortic Valve**	Male (vs Female)	1.16 (0.52–2.58)	0.720	1.16 (0.35–3.91)	0.808
	Age (years)	1.02 (0.99–1.05)	0.173	0.99 (0.94–1.03)	0.588
	Hypertension	0.56 (0.24–1.35)	0.198	1.49 (0.41–5.40)	0.541
	Diabetes Mellitus	0.00 (0.00–inf)	0.999	0.00 (0.00–inf)	0.999
	Chronic Kidney Disease	0.20 (0.03–1.57)	0.125	0.00 (0.00–inf)	1.000
	Heart Failure	3.12 (1.27–7.64)	0.013	0.52 (0.06–4.22)	0.537

Values are presented as odds ratios (ORs) with 95% confidence intervals (CIs) from multivariable logistic regression models in the overall cohort and genetically positive (GP) subgroup, adjusted for age, hypertension, diabetes mellitus, chronic kidney disease, and heart failure. Model sample sizes were 783/353 for moderate–severe aortic regurgitation, moderate–severe tricuspid regurgitation, moderate–severe mitral regurgitation, and mitral valve prolapse, and 782/353 for bicuspid aortic valve (overall cohort/GP subgroup).

S4F. Cardiac Surgery

Outcome	Predictor	OR (95% CI)	p value	GP OR (95% CI)	GP p value
**Aortic Root Replacement or Repair**	Male (vs Female)	2.47 (1.81–3.37)	< 0.001	2.63 (1.65–4.19)	< 0.001
	Age (years)	1.01 (1.00–1.02)	0.342	1.00 (0.98–1.01)	0.674
	Hypertension	2.20 (1.59–3.05)	< 0.001	3.15 (1.90–5.21)	< 0.001
	Diabetes Mellitus	0.79 (0.39–1.58)	0.507	0.64 (0.19–2.16)	0.470
	Chronic Kidney Disease	1.76 (1.01–3.07)	0.048	3.62 (1.36–9.65)	0.010
	Heart Failure	3.47 (2.16–5.59)	< 0.001	2.48 (1.29–4.76)	0.006
**Aortic Valve Surgery**	Male (vs Female)	2.10 (1.52–2.89)	< 0.001	1.87 (1.15–3.04)	0.012
	Age (years)	1.02 (1.01–1.03)	< 0.001	1.01 (1.00–1.03)	0.135
	Hypertension	1.73 (1.25–2.40)	< 0.001	2.11 (1.27–3.50)	0.004
	Diabetes Mellitus	0.61 (0.31–1.19)	0.145	0.45 (0.14–1.46)	0.186
	Chronic Kidney Disease	2.02 (1.21–3.35)	0.007	2.81 (1.26–6.23)	0.011
	Heart Failure	3.02 (1.98–4.60)	< 0.001	2.91 (1.61–5.27)	< 0.001
**Mitral Valve Surgery**	Male (vs Female)	1.14 (0.76–1.72)	0.524	0.68 (0.36–1.28)	0.232
	Age (years)	1.00 (0.99–1.01)	0.956	1.02 (0.99–1.04)	0.165
	Hypertension	0.66 (0.42–1.02)	0.062	0.35 (0.17–0.74)	0.006
	Diabetes Mellitus	0.50 (0.18–1.37)	0.175	0.00 (0.00–inf)	0.999
	Chronic Kidney Disease	0.83 (0.44–1.55)	0.557	1.01 (0.39–2.65)	0.984
	Heart Failure	6.04 (3.79–9.62)	< 0.001	8.50 (4.08–17.71)	< 0.001

**Tricuspid Valve Surgery**	Male (vs Female)	0.97 (0.38–2.48)	0.952	0.85 (0.23–3.18)	0.813
	Age (years)	0.99 (0.96–1.02)	0.522	1.01 (0.96–1.06)	0.752
	Hypertension	0.66 (0.24–1.84)	0.429	0.41 (0.09–1.79)	0.236
	Diabetes Mellitus	1.54 (0.31–7.61)	0.600	0.00 (0.00–inf)	1.000
	Chronic Kidney Disease	1.90 (0.64–5.68)	0.251	1.92 (0.41–9.03)	0.410
	Heart Failure	7.37 (2.75–19.77)	< 0.001	11.89 (2.67–53.01)	0.001

Values are presented as odds ratios (ORs) with 95% confidence intervals (CIs) from multivariable logistic regression models in the overall cohort and genetically positive (GP) subgroup, adjusted for age, hypertension, diabetes mellitus, chronic kidney disease, and heart failure. Model sample sizes were 783/353 for aortic root replacement or repair, aortic valve surgery, mitral valve surgery, and tricuspid valve surgery (overall cohort/GP subgroup).
